# “I Speak Up for Myself, My Family, and for My Community”: How Asian Americans, Native Hawaiians, and Pacific Islanders Respond to Anti‐Asian Racism During COVID‐19

**DOI:** 10.1002/jcop.23160

**Published:** 2024-11-20

**Authors:** Julie K. Nguyen, Wendy Chu, Stephanie H. Yu, Andrea C. Ng

**Affiliations:** ^1^ Department of Psychology University of South Carolina Columbia South Carolina USA; ^2^ Department of Psychiatry and Behavioral Sciences University of California, San Francisco San Francisco California USA; ^3^ Department of Psychology University of Hawai'i at Manoa Honolulu Hawaii USA

**Keywords:** Asian American, coronavirus, qualitative, racial discrimination

## Abstract

This qualitative study explored how Asian Americans, Native Hawaiians, and Pacific Islanders (AANHPIs) responded to anti‐Asian racism during COVID‐19. Participants (*n* = 459; *M*_age_ = 26.4 years; 77.8% female) completed a survey and responded to the open‐ended question: “How have you responded to anti‐Asian racism since the beginning of the COVID‐19 pandemic?” Reflective thematic analysis was utilized to code responses. Three themes were identified, indicating that AANHPIs engaged in knowledge sharing and cultural exploration; experienced emotional reactions that galvanized efforts to take care of themselves, friends, family, and their community; and engaged in advocacy and activism to advance social justice. Subthemes were also identified to further characterize responses. Results reflect how AANHPI communities responded to anti‐Asian racism by leveraging cultural strengths and taking the initiative to create social change. These findings have individual‐, organizational‐, and community‐level implications to mitigate the impacts of anti‐Asian racism and promote the well‐being of AANHPI communities.

The COVID‐19 pandemic brought abrupt change to daily life and exposed the United States' longstanding structural inequities driven by racism, initiating our society's racial reckoning. For Asian Americans, Native Hawaiians, and Pacific Islanders (AANHPIs), reports of discrimination and hate incidents surged drastically during the COVID‐19 pandemic, suggesting that AANHPIs were at the intersection of a syndemic, or synergy of co‐occurring and compounding epidemics (Saw et al. 2022). To highlight, a report by Stop AAPI Hate documented over 11,000 hate incidents from March 2020 to March 2022 (Stop Asian American and Pacific Islander Hate 2022). Early in the pandemic, targeted and violent acts of racism often made media headlines representing harrowing experiences—from an 84‐year‐old Thai American immigrant who was killed after being shoved to the ground during a morning walk, to a Myanma father and his 6‐year‐old son who were attacked while grocery shopping, to the Atlanta‐area spa shootings that killed four Korean women, two Chinese women, and two White people. Negative and xenophobic rhetoric toward AANHPIs (e.g., referring to COVID‐19 as the “China virus” or “Kung flu”) was also blatantly popularized by high‐ranking politicians. For many, the pervasiveness of COVID‐related anti‐Asian discrimination brought to light the realities of interpersonal, institutional, and structural racism toward AANHPIs that has persisted within the United States for centuries (see Stanford Libraries n.d., for historical context).

Experiences of anti‐Asian racism, whether direct, vicarious, overt, or subtle, have profound implications on the health and well‐being of AANHPIs. For example, Gee et al.'s (2009) review found that self‐reported discrimination among Asians or Pacific Islanders was associated with poorer physical functioning, risky health behaviors, and most commonly, poorer mental health outcomes. Yu et al.'s (2024) study also documented the harrowing impacts of anti‐Asian racism through qualitative inquiry. Experiences of anti‐Asian racism may also be compounded for individuals who hold intersectional minoritized identities (Azhar et al. 2021; Sue et al. 2007) and may be influenced by cultural factors such as racial socialization (Alvarez, Juang, and Liang 2006) and acculturation (Yu et al. 2024), leading to varying experiences of anti‐Asian racism. Research suggests that COVID‐19‐related anti‐Asian discrimination has been associated with greater symptoms of posttraumatic stress (Hahm et al. 2021), anxiety (Cheah et al. 2020), and depression (Woo and Jun 2021). With the staggering increase of racist acts against AANHPIs since the COVID‐19 syndemic, these studies have contributed to our collective knowledge of the detrimental impacts of racism on AANHPIs.

Less research has explored how AANHPIs respond to experienced racism. There have been some proximal efforts examining AANHPIs' immediate responses after experiencing a racist event. For instance, a study conducted by Oh and Litam (2022) found that internal and individualistic coping responses, such as problem‐focused coping (e.g., I make a plan of action and follow it), moderated the relationship between experienced discrimination and poor mental health. Some research has also explored more collectivistic responses that seek to combat the effects of racism and advance a group's well‐being (e.g., Tao et al. 2024; Wei et al. 2010). Collective responses may include activist behaviors (e.g., voting) and social media engagement (e.g., spreading awareness of social issues). One study by Tao et al. (2024) explored social media activism as a collective response to racism during the COVID‐19 pandemic and found that Asian Americans who engaged in more problem‐focused coping were also more likely to participate in social media activism. Another study by Wei et al. (2010) found that collective coping (e.g., leveraging family support) moderated the relationship between racial discrimination stress and depressive symptoms, illustrating how a response that centers collectivism can influence mental health. Overall, these studies reflect the heterogeneity in how AANHPIs respond to racism.

However, research exploring AANHPIs' responses to racism has primarily focused on individualistic (e.g., independent actions centered on taking care of oneself) and reactive (e.g., tend to immediate problems) responses. This reflects a dominant Eurocentric perspective and depreciates responses that are more collectivistic and proactive in nature. To address this, scholars have advocated for allowing the global majority to define the ways in which they respond to racism, as opposed to using pre‐defined lists of coping strategies and individualistic actions to capture their responses (Lee et al. 2023). Moreover, they describe the need for healing research that considers how marginalized communities examine sociocultural conditions and move toward liberatory change through collective resistance, rest, reflection, and rejoice, as these responses can reflect healing from racism (Lee et al. 2023). By allowing AANHPIs to describe the ways in which they choose to respond to anti‐Asian racism, we can better understand the different actions that AANHPIs may take to combat racial oppression in their lives and community. Inspired by scholars who have championed advancing more culturally robust terminology and concepts (French et al. 2020), we use the word “response” to encapsulate both the collectivistic, proactive actions (akin to resistance) and the individualistic, reactive actions (akin to coping) that communities of color may take to resist racism.

Toward the goal of advancing scholarship that promotes healing in AANHPI communities affected by anti‐Asian racism, this study aimed to address two gaps in the extant literature. First, while studies that document the deleterious impacts of racism on AANHPIs are certainly needed, studies documenting AANHPIs' responses to racism are equally important. To our knowledge, there has been only one preliminary investigation on this topic (Chiang 2020). Second, given that Chiang () largely used quantitative methods of documenting responses, there is an opportunity for qualitative research that examines AANHPIs' response to anti‐Asian racism. Employing a qualitative approach enhances our ability to capture nuanced experiences while amplifying community members' lived experiences. By harnessing community‐derived and cultural knowledge from AANHPIs' responses to anti‐Asian racism, this work holds translational value for informing culturally grounded programs and interventions to support AANHPIs in resisting and healing from anti‐Asian racism. Given the qualitative nature of this study, no a priori hypotheses were defined.

## 1 Methods

The present study used data from a national online survey for AANHPIs that included multiple questionnaires and open‐ended questions. Data were collected about one and a half years after the beginning of the COVID‐19 pandemic (November 2021 to January 2022) and during a time when there were high‐profile targeted, violent, and fatal acts of racism against AANHPI individuals. Purposeful sampling was employed for recruitment. A flyer with a survey link was distributed to AANHPI‐focused organizations, centers, universities, listservs, and media platforms. Participants were eligible if they were at least 18 years of age, identified as AANHPI, and could read and write in English, Chinese, and/or Vietnamese. Informed consent was gathered at the beginning of the survey, which could be completed on any internet‐connected device. Participants received a $5 gift card for survey completion. Following a qualitative paradigm, we focused on the open‐ended question, “How have you responded to anti‐Asian racism since the beginning of the COVID‐19 pandemic?” that was placed toward the end of the survey. Participants typed their response in a textbox with no word limit. Study procedures and materials were approved by the Institutional Review Board of the University of South Carolina.

### 1.1 Participants

Demographics are presented in Table 1. Participants consisted of 459 AANHPI adults whose age ranged from 18 to 72 years (*M* = 26.6, *SD* = 9.5). Of the participants who responded, most identified as cisgender women (*n* = 355, 77.5%), followed by cisgender men (*n* = 88, 19.2%) and gender expansive (*n* = 15; 3.3%). Participants identified with one ethnic group (*n* = 375, 81.7%) or multiple ethnic groups (*n* = 84, 18.3%). Ethnic representation from greatest to least included Chinese, Vietnamese, Filipino, Korean, Taiwanese, Indian, Japanese, Hmong, White, Cambodian, Native Hawaiian, Indonesian, Pakistani, Hispanic, Malaysian, Singaporean, Thai, Bengali, Laotian, Sri Lankan, Tongan, Manchurian, Mien, Montagnard, Okinawan, Punjabi, and Samoan. The “White” category was added based on write‐in responses, as some participants specified multiracial identity. Using the US geographical categorizations described by Hughes et al. (2022), most participants lived in the West (*n* = 198, 43.1%), followed by the Northeast (*n* = 96, 21.0%), South (*n* = 94, 20.4%), and Midwest (*n* = 71, 15.1%) at the time of survey completion. Of those who responded, the modal annual household income was between $50,000 and $100,000 (*n* = 121, 29.3%) and the most common highest educational degree attained was a high school degree (*n* = 175, 38.1%). Finally, of the participants who provided their generation status, most identified as second generation (born in the United States with at least one parent born outside the United States; *n* = 276, 61.1%), followed by first generation (born outside the United States; *n* = 136, 30.1%), and third generation and higher (born in the United States with two parents and at least grandparent born in the United States; *n* = 40, 61.1%).

**Table 1:** Participant demographics.

Demographic variable	Value
Age (*N* = 459)	*M* (*SD*)
Age in year	26.6 (9.5)
Gender (*N* = 458)	*n* (%)
Cisgender woman	355 (77.5)
Cisgender man	88 (19.2)
Nonbinary	8 (1.8)
Gender nonconforming	5 (1.1)
Gender fluid	1 (0.2)
Transgender woman	1 (0.2)
Ethnicity (*N* = 459)	*n* (%)
*East Asian*	307 (66.9)
Chinese	177 (38.6)
Korean	48 (10.5)
Taiwanese	44 (9.6)
Japanese	36 (7.8)
Okinawan	1 (0.2)
Manchurian	1 (0.2)
*Southeast Asian*	169 (22.7)
Vietnamese	67 (14.6)
Filipino	57 (12.4)
Hmong	25 (5.4)
Cambodian	8 (1.7)
Laotian	2 (0.4)
Indonesian	5 (1.1)
Thai	3 (0.7)
Mien	1 (0.2)
Montagnard	1 (0.2)
*South Asian*	53 (11.6)
Indian	37 (8.1)
Pakistani	5 (1.1)
Malaysian	3 (0.7)
Singaporean	3 (0.7)
Bengali	2 (0.4)
Sri Lankan	2 (0.4)
Punjabi	1 (0.2)
*Hawai'ian and Pacific Islander*	10 (2.1)
Native Hawaiian	7 (1.5)
Tongan	2 (0.4)
Samoan	1 (0.2)
*Other*	16 (3.5)
*Hispanic*	3 (0.7)
*White*	13 (2.8)
Location (*N* = 459)	*n* (%)
West	198 (43.1)
Northeast	96 (21.0)
South	94 (20.4)
Midwest	71 (15.5)
Income (*N* = 413)	*n* (%)
Less than $25,000	63 (15.3)
$25,000–$50,000	88 (21.3)
$50,000–$100,000	121 (29.3)
$100,000–$200,000	89 (21.5)
More than $200,000	52 (12.6)
Education (*N* = 452)	*n* (%)
High school degree or lower	175 (38.1)
Associate/trade degree	37 (8.1)
Bachelor's degree	129 (28.1)
Master's degree	75 (16.3)
Doctoral degree	43 (9.4)
Generation (*N* = 452)	*n* (%)
First generation	136 (30.1)
Second generation	276 (61.1)
Third‐generation and higher	40 (8.8)

*Note:* Participants could identify with one or more racial/ethnic identities; thus, percentages sum to greater than 100%.

### 1.2 Data Analysis

We used reflexive thematic analysis to analyze participant responses (Braun and Clarke 2019, 2021). Reflexive thematic analysis emphasizes theoretical knowingness and transparency, and fully acknowledges the roles and experiences of the researchers on the subjective interpretation and construction of themes. As such, we, the coding and analysis team, articulate our positionalities at the time of the study. The first author is a cisgender woman, daughter of Vietnamese immigrants, and PhD student in clinical‐community psychology. The second author is a cisgender woman, daughter of low‐income Chinese immigrants from a large metropolitan city, and PhD candidate in clinical‐community psychology. The third author is a cisgender woman, daughter of Chinese/Taiwanese immigrants, and PhD candidate in clinical psychology. The fourth author is a cisgender woman, first‐generation Malaysian Chinese immigrant, and PhD student in clinical psychology. With our identities reflected in the research question and sample, we discussed how participants' experiences impacted, resonated, and/or differed from our ow personal experiences and perspectives throughout the entire research process, especially during the coding and qualitative analysis process. For example, we noticed how our experiences as clinical psychology trainees shaped our ability to see emotional reactions, how our held values in social justice and equity aligned with some participants' actions, and how our experiences in urban liberal cities differed from participant experiences in rural conservative areas.

Led by the first and second author, the full coding team (all authors) followed a six‐phase analytic process (Braun and Clarke 2006, 2021). First, the coding team read all participants' responses to the open‐ended question “How have you responded to anti‐Asian racism since the beginning of the COVID‐19 pandemic?” to gain familiarity with the data. Second, 28 initial codes that described features within the data were developed through inductive discussions of the responses. Coders then systematically categorized responses into these codes, allowing for responses to be categorized into multiple codes if suitable. New codes were added iteratively throughout the coding process if appropriate. Upon completion, coders reviewed the collated responses within each code as a data engagement step and collapsed/created new codes as necessary. Coders met regularly via Zoom to discuss our experiences and reflection in coding and addressed any challenges in categorizing responses. Third, a thematic map was created via PowerPoint to visualize relationships between codes (e.g., codes were entered into text boxes and similar codes were placed in proximity to each other). At this phase, initial subthemes and overarching themes were constructed using the visual map as a guide (e.g., themes were written in bordered text boxes that described the pattern of similar codes). Fourth, coders further discussed and reviewed all themes to ensure the “overall story” of the data was reflected (Braun and Clarke 2006). The fifth and sixth phases of analysis were interwoven, as themes and subthemes were initially refined and named, and the writing of the report informed further refinement of themes. Theme generation and analysis were guided by a critical theory‐informed research paradigm to recognize the societal and historical implications on constructed experiences and the dialectical relationship between oppression and social action (Ponterotto 2005). Of note, in this study, coders primarily sought to analyze data and generate themes based on the collective sample of AANHPI responses, as opposed to dividing responses based on sociodemographic variables (e.g., by ethnic group). While this allowed us to infer shared collective experiences from a heterogenous sample, there are drawbacks of this approach, which are discussed in Section 3.1.

## 2 Results

We identified three themes and eight subthemes, which are summarized in Table 2.

**Table 2:** Summary of themes and subthemes.

Theme	Subtheme
1: AANHPIs engaged in knowledge sharing and cultural exploration.	1.1: Sharing information and resources served to build community resistance.
	1.2: Learning about and connecting to AANHPI culture fostered cultural authenticity.
2: Emotional reactions, particularly fear, anxiety, and hypervigilance, galvanized efforts to take care of themselves, others, and their community.	2.1: AANHPIs protected and took care of oneself primarily by engaging in safety behaviors.
	2.2: Concern for loved ones and one's community led to collective caretaking.
	2.3: The act of ignoring anti‐Asian racism can be a form of resistance.
3: AANHPIs engaged in advocacy and activism in response to anti‐Asian racism.	3.1: AANHPIs became agents of change on individual, structural, and civic levels.
	3.2: The experience of oppression fosters solidarity with other marginalized communities.
	3.3: AANHPIs engaged in conscious philanthropy to support Asian organizations and businesses.

### 2.1 Theme 1: AANHPIs Engaged in Knowledge Sharing and Cultural Exploration

#### 2.1.1 Subtheme 1.1: Sharing Information and Resources Served to Build Community Resistance

Participants described sharing posts to raise awareness of anti‐Asian racism, which was largely motivated by wanting to “educate others” on the prevalence and severity of anti‐Asian racism and to inform AANHPIs on how to navigate harmful situations. To highlight, a 19‐year‐old second‐generation Vietnamese woman and a 19‐year‐old first‐generation Native Hawaiian woman wrote, respectively,

I have reposted on my Instagram story about the racism so everybody in the Asian community can be careful.

I would spread awareness on the anti‐Asian racism news in my social media (Instagram, Snapchat, etc.) so people know that this is going on in the world and needs to be addressed.

Sharing information also seemed to be connected to increasing recognition of AANHPI communities and building resistance. A 21‐year‐old first‐generation Cambodian man said,

I have also connected with other AAPI groups to share resources and connect to each other. We all need each other. And we need the other communities to see us… We also need to change within our own communities to stand up and not be afraid.

These responses highlight how AANHPIs used knowledge as a form of social mobilizing, which enabled their community to be educated, prepared, and seen. In this sample, social media was a helpful tool for various purposes and to reach different audiences (e.g., educate individuals who are non‐Asian or may not be aware of anti‐Asian racism, inform AANHPI community how to stay safe). Resource disseminating and resource mapping was a collectivistic‐oriented action to ensure others possessed the knowledge to enhance individual and community wellness. It may have also supported AANHPIs' sense of self‐efficacy and agency through participation in digital movements. Further, knowledge sharing facilitated connection and communication during a time when isolation was a necessity for the COVID‐19 pandemic.

#### 2.1.2 Subtheme 1.2: Learning About and Connecting to AANHPI Culture Fostered Cultural Authenticity

Participants also engaged in self‐exploration to gain knowledge and connect with their AANHPI culture. For example, staying “up to date” with news and AANHPI‐relevant issues, and learning more about AANHPI history through “readings” and “documentaries” helped participants feel “more educated” and “aware.” Participants described the restorative outcomes of contacting cultural knowledge and cultural roots. As a 19‐year‐old second‐generation Vietnamese woman shared,

Consuming art, food, and history about my ethnic group has been healing.

For some, connecting to culture took the form of joining Asian identity specific affinity groups. These groups not only allowed participants to have a “safe space” to discuss racial‐ethnic issues, but it also allowed them to collectively work toward shared values, missions, and goals. For example, a 24‐year‐old first‐generation Japanese and Native Hawaiian woman said,

I joined an Asian group that aims towards mentoring other Asians. From there, I created illustrations with mental health resources for Asian Americans specifically and other social justice initiatives for other Asian Americans and Pacific Islander to look at.

Participants highlighted a diversity of responses that allowed them to connect to their cultural heritage. These actions deepened their critical understanding of historical and contemporary issues affecting AANHPIs and encouraged social connection and community with others. Resource and knowledge sharing, as mentioned in the previous subtheme, likely played a role in spreading opportunities to learn about one's culture (e.g., sharing a recommended book list on social media). Moreover, given the diversity of the sample, the level of cultural exploration was likely influenced by personal awareness, understanding, and experiences. For example, consuming literature on AANHPI issues may have been a response for participants who have never been exposed to AANHPI history, inferring that leaning into one's AANHPI identity and culture may be a “healing” response.

### 2.2 Theme 2: Emotional Reactions, Particularly Fear, Anxiety, and Hypervigilance, Galvanized Efforts to Take Care of Themselves, Others, and Their Community

#### 2.2.1 Subtheme 2.1: In Times of Violence, AANHPIs Protected and Took Care of Oneself Primarily by Engaging in Safety Behaviors

Participants shared that their emotional reactions, particularly fear and anxiety, prompted self‐care in the form of defensive or safety behaviors. Participants described “avoiding going out” in public, shrinking themselves—at times concealing their AANHPI identity—to reduce interactions with others, and remaining “hyper‐aware” of their surroundings “to not get discriminated against.” A 27‐year‐old above third‐generation Vietnamese woman stated,

I have been a bit more careful about going outside but also wearing a mask helps conceal the fact that I am Asian.

Indeed, these safety behaviors coincided with safety concerning the COVID‐19 pandemic. A 30‐year‐old first‐generation Chinese and Malaysian woman shared,

I stay away from scary place and I stay on the farm to work if I don't need to go anywhere. Too much Covid and hate.

Some individuals even purchased “self‐defense” weapons such as pepper spray and “hosted self‐defense classes.” The overbearing emotional toll from anti‐Asian racism during the early parts of the COVID‐19 pandemic led some participants to seek a “psychiatrist” or “therapist.”

These responses highlighted the impact of AANHPI‐directed violence and discrimination that is prevalent in many US communities, which instilled fear, threatened participants' livelihood, and necessitated defensive and safety actions. These responses were complexly tied to sociocultural realities of the ongoing scapegoating of AANHPIs, particularly Chinese Americans, during the COVID‐19 pandemic, as some participants felt it was necessary to erase and minimize their racial identity from being perceived by others. Some of these emotions and reactions, such as isolation, avoidance, and hypervigilance, may be indicative of trauma symptoms.

#### 2.2.2 Subtheme 2.2: Concern for Loved Ones and One's Community Led to Collective Care Taking

Participants described having anxiety and concerns for their community, at times describing greater care and concern for their children, parents, friends, and elders than for themselves. A 24‐year‐old second‐generation Cambodian and Vietnamese man expressed,

I'm always checking in on my mom as well because I'm terrified someone will harm her.

Participants described “holding space” for one another to “process” ongoing anti‐Asian racism through conversations and discussions, “reaching out” to provide and receive social and emotional support in their community, and “reminding” loved ones to protect themselves with self‐defense tools and to be aware of their surroundings. A 20‐year‐old second‐generation Chinese and Korean woman described their efforts toward “being a safe space to talk about anti‐Asian racism” and “sharing information and safety protocols to elders and parents.” A 35‐year‐old second‐generation Chinese woman said,

I've been able to talk to my friends (Asian and non‐Asian) about the tragic events that have happened to the Asian community and discuss our feelings. These conversations were very meaningful to me and served as a form of support and stress relief.

These actions reflect a close connection to kin and the importance of protecting those around us. These actions also included sharing knowledge and creating safe spaces, which have been mentioned in prior subthemes. Notably, there was a strong emphasis on caring responses that involved intergenerational relationships, specifically parents and elders. For some participants, these collectivistic responses were of greater priority than individualistic responses. It is likely that “checking in” with loved ones during a time of racial turmoil also increased communication and had a positive effect on participants by alleviating worries and fears.

#### 2.2.3 Subtheme 2.3: The Act of Ignoring Anti‐Asian Racism Can Be a Form of Resistance

Some participants described how they would “ignore” situations and “walk away,” likely referring to if a perpetrator made a racist comment to them. Other participants explicitly identified ignoring as an act of resistance in combating anti‐Asian racism. For example, a 36‐year‐old second‐generation Chinese man stated,

I've just tried to live life normally and not let it affect me. Doing so would feel like giving in to them.

By not enabling racism to dictate their actions and emotional reactions, participants expressed being able to live their lives on their own terms despite anti‐Asian racism. For instance, a 24‐year‐old second‐generation Japanese woman said,

I found [anti‐Asian racism] repulsive and realized that I didn't/shouldn't really care what anti‐Asian racists thought and I should just live my life without fearing them.

This emboldened stance described by participants seemed to be an active and adaptive response to not succumb to the effects of racial oppression, thereby allowing them to live more freely. It may also be a specific individual‐level coping mechanism to protect themselves from negative or unwanted emotional reactions. At the same time, it is unclear whether ignoring AANHPI racism could be related to denying the existence of racism altogether, experiencing of avoidance, or perceiving a lack of agency to intervene in racist encounters.

### 2.3 AANHPIs Engaged in Advocacy and Activism in Response to Anti‐Asian racism

#### 2.3.1 Subtheme 3.1: AANHPIs Became Agents of Change on Individual, Structural, and Civic Levels

In response to anti‐Asian racism, AANHPIs were motivated to create positive change in their communities through a multitude of ways. Participants shared how they joined AANHPI advocacy groups that promoted “diversity, equity, and inclusion” in their professional and academic contexts. Some took initiative to lead “healing spaces” in their communities and facilitated other events to promote collective well‐being and advance advocacy efforts. A 38‐year‐old first‐generation Filipino woman stated,

I was lucky to work in a department with a larger number of Asian‐identified folks providing mental health services. We processed our reactions together, organized processing space for other Asian folks in the university, and seminar about Asian experiences and how to better support us in mental health spaces. I also started therapy again to process the impacts of these cumulative events on my own mental health.

Participants also engaged in a range of sociopolitical and civic activities such as assisting other AANHPIs “to become registered voters,” “attending protests,” “signing petitions,” and speaking or writing to “officials” who hold power at the local, state, and federal level. AANHPIs also described efforts to be more of an “upstander” by “speaking up” and calling out racism when they witness it. A few individuals shared how they made significant careers changes to support their community. A 45‐year‐old second‐generation Vietnamese woman shared,

I changed jobs to an AANHPI related organization to help promote the arts and resources for AAPIs.

These responses illustrate a clear connection between witnessing anti‐Asian racism and creating real‐world social change to improve the lives affected by racism. Participants' emotional reactions and concern for others culminated in a motivation to address the conditions that affect their communities through sociopolitical and civic engagement. Moreover, responses noted earlier, such as joining social groups and sharing knowledge, may strengthen AANHPIs' desire to enact collective change. Indeed, these responses fit within the broader context of a growing social movement to act and end racial violence toward AANHPIs and promote AANHPI well‐being in a disconcerting time.

#### 2.3.2 Subtheme 3.2: The Experience of Oppression Fosters Solidarity With Other Marginalized Communities

Participants shared that awareness of anti‐Asian hate led them to recognize that White supremacy not only impacts AANHPIs but also other marginalized groups, resulting in action and solidarity with other marginalized communities. A 39‐year‐old second‐generation Hmong woman and a 26‐year‐old second‐generation Chinese woman shared, respectively,

I work incrementally toward supporting dialogue and solidarity among communities of color.

I try to do my part to look beyond anti‐Asian racism to more pressing issues such as intersectionality with other minorities and systemic injustice that disproportionately affects other groups.

In particular, several participants described speaking up about anti‐Blackness by “opening up conversations” about anti‐Blackness in the Asian community and “correcting” AANHPIs when they express anti‐Black sentiments.

These responses reflected a critical understanding of a shared common experience of oppression among marginalized communities and an awareness of barriers that have been placed to divide communities. Specifically, AANHPIs noted an awareness of the issue of racial triangulation and the anti‐Black perspectives it harbors. These responses are tied to how AANHPIs recognize the need to engage in individual, structural, and civic change, as discussed, not only to advance the well‐being of one's own community but also of allied communities. These responses that center solidarity reflect a sense of collective caretaking that extends beyond the AANHPI community.

#### 2.3.3 Subtheme 3.3: AANHPIs Engaged in Conscious Philanthropy to Support Asian Organizations and Businesses

Participants engaged in philanthropy to support AANHPI‐owned organizations and businesses during a time when many suffered financial consequences from anti‐Asian hate. This included “donating money” to AANHPI organizations and to “support victims of hate crimes.” Participants mentioned other philanthropic acts such as volunteering their time or linguistic abilities to support fundraisers or attending events to raise more awareness about discrimination against AANHPIs. To illustrate, a 25‐year‐old second‐generation Chinese woman and a 22‐year‐old second‐generation Filipino man stated, respectively,

I try to support local, Asian businesses and have supported more AANHPI‐owned businesses through online means.

Besides donate here and there, I haven't done much actually. I definitely want to, but feel exhausted and don't have the energy to do so.

While different forms of philanthropy were noted across many responses, donating financial resources was particularly common. Not only has making donations become easier with online pages, but donating may have been a more accessible response to counter the impact of anti‐Asian racism. For instance, responses such as participating in protests require time and energy that some AANHPIs may not have the luxury of and may even put oneself in danger by potential interactions with law enforcement. Overall, the range of philanthropic acts demonstrates how AANHPIs were committed to supporting their collective community.

## 3 Discussion

This study explored how AANHPIs responded to anti‐Asian racism in the context of the COVID‐19 pandemic. Based on responses from an ethnically diverse sample of AANHPIs, we identified three themes and eight subthemes that reflected a range of individual and collective actions. Often motivated by an intolerance to racial injustice, these actions fostered cultural authenticity, took care of the self and others, and promoted advocacy/activism. Together, these actions suggest that AANHPIs are taking initiative in ways that promote their own and collective healing in response to anti‐Asian racism. We discuss these patterns in relation to psychological and community constructs that may inform multilevel interventions.

AANHPIs' responses to racism reflected varying aspects of critical consciousness. Critical consciousness—originating from theories presented in Paulo Freire's *Pedagogy of the Oppressed*—has been defined as one's capacity for “critical analysis of their social conditions and individual or collective action taken to change perceived inequities” (Diemer et al. 2017, p. 462; Freire 1970). Developing critical consciousness is thus central to community liberation and healing from racial oppression (French et al. 2020). Across responses, AANHPIs described engaging in both critical reflection and critical action (Diemer et al. 2017); whereas critical reflection involves analysis of oppressive forces and rejection of social inequities, and critical action involves “moving from commitment to action” toward sociopolitical change (French et al. 2020, p. 26). In terms of critical reflection, participants described how experiences of anti‐Asian racism acted as a catalyst for recognizing and acknowledging White supremacy and systemic injustice. Participants also described critical reflection through building cultural self‐knowledge, alongside exploration of both historical and current events related to anti‐Asian racism. Contrary to stereotypes of AANHPIs as apolitical (Chan 2021), participants took critical action in the form of advocacy, including participating in protests, signing petitions, writing to government officials, joining advocacy groups, and creating community healing spaces. In addition, participants described how experiences of anti‐Asian racism motivated them to reflect and challenge injustice against (e.g., xenophobia) and within (e.g., anti‐Blackness) their own community, and uplift other marginalized communities (e.g., people who are disabled, other communities of color). These findings align with prior research that highlights the diverse forms of AANHPI sociopolitical participation and the mobilizing power within AANHPI communities to advance collective liberation for all (Lien et al. 2001; Lei, Frazer‐Klotz, and Szanton 2022; Wang and Santos 2023). Indeed, community psychology as a field has emphasized the role of advancing social justice in community's well‐being, with research supporting this relationship particularly in the context of resisting racial oppression (Maker Castro, Dull, et al. 2022, Maker Castro, Wray‐Lake, and Cohen 2022).

Findings suggest that AANHPIs tapped into and engaged with their community's cultural wealth as a response to racism (Yosso 2005). Community cultural wealth is a concept proposed by Yosso (2005) that counters the notion that marginalized communities (e.g. first‐generation students of color) have deficits which impede their ability to thrive and succeed under challenging circumstances (e.g., navigating higher education). This model posits that marginalized communities possesses six forms of capital: aspirational (e.g., ability to maintain hopes and dreams despite present circumstances), navigational (e.g., skills of maneuvering through institutions), social (e.g., networks of people and community resources), linguistic (e.g., skills gained through communication experiences in different styles), familial (e.g., knowledge and practices nurtured through connection to kin), and resistant (e.g., knowledge and skills fostered through oppositional behavior that challenges inequity) capital. This wealth reflects accumulated cultural knowledge, skills, abilities, and contacts that communities have utilized to combat oppression (Yosso 2005). AANHPIs in our sample especially drew on their familial and social capital in response to anti‐Asian racism (Yosso 2005). In terms of familial capital, AANHPIs connected with their familial/kin's cultural heritage by joining Asian affinity groups and intentionally immersing themselves in AANHPI knowledge (e.g., via shows, art, music, reading) in the face of anti‐Asian racism. This capital allows an individual to engage in cultural authenticity through honoring their ancestral roots, wisdom, and teachings (French et al. 2020), which has been associated with better mental health outcomes in AANHPI youth (Luthar, Ebbert, and Kumar 2021). Moreover, this sense of connection to kin may facilitate a sense of community and belongingness, which can be a protective factor (Zhou et al. 2012). In terms of social capital, AANHPIs described seeking community and engaging in collective caretaking. This took on many forms, including reaching out to friends, processing emotional reactions to racism with family members, identifying “safe spaces” (e.g., Asian identity affinity groups), and supporting Asian‐owned businesses. The gaining and giving of social capital and putting the needs of others above oneself reflects a sense of collectivism, which is a prominent value across many AANHPI cultures (Kaholokula, Okamoto, and Yee 2019; McLean et al. 2021; Sue and Sue 2015). Overall, these responses illustrate the value of community connection and involvement.

Finally, AANHPIs' responses demonstrated resistance, another source of community capital. For some individuals, resistance took the form of choosing to persevere and continue with life as normal despite anti‐Asian racism in everyday life, which can be protective for some (Ellefsen, Banafsheh, and Sandberg 2022), but could also be indicative of denial of anti‐Asian racism (Yi et al. 2023). AANHPIs also resisted the threat of physical safety by protecting themselves (e.g., carrying a taser or pepper spray) and by protecting elders. For example, there have been on‐the‐ground efforts, such as Compassion in Oakland, to accompany Asian elders in Oakland's Chinatown. AANHPI social workers have also advocated for resources and resources to empower older adults (Lee et al. 2023). Moreover, AANHPIs resisted the threat of silencing by leveraging social media to amplify their voice, which is a form of digital community resistance (Simpson, Napawan, and Snyder 2019; Wilson and Starbird 2021). For instance, many participated in sharing hashtags such as #StopAAPIHate and disseminating resources that may promote others' resistance. Aligned with community psychology values, understanding how AANHPIs engage in resistance to systematic and structural issues such as racial oppression is key to elevating liberatory behaviors that can empower marginalized communities (Watts and Serrano‐García 2003). Indeed, community psychologists have called for the need to attend to and promote “everyday resistance” in marginalized communities of color (Rosales and Langhout 2020).

### 3.1 Limitations

Findings must be considered in the context of several limitations. AANHPIs provided their responses through an open‐ended question embedded within a larger survey. Although the survey format allowed for larger reach and accessibility, responses collected through an interview format may have garnered more in‐depth responses. Survey fatigue may have also influenced responses. Furthermore, the survey was limited to AANHPIs over the age of 18 and those who could complete the survey in English, Chinese, or Vietnamese; thus, these findings may not generalize to AANHPI youths and those whose language and literacy was beyond the ones mentioned. Due to the online nature of the survey, the sample may have been younger and may have excluded AANHPIs without reliable internet access. As flyers were distributed through AANHPI‐centered groups, the sample may reflect AANHPIs who were already more engaged and responsive to social issues. Data collection was also conducted approximately one and a half years after the initial onset of the COVID‐19 pandemic in 2020, and thus does not capture immediate responses to anti‐Asian racism during the onset; that said, this enabled us to understand how AANHPIs responded to an accumulation of rising anti‐Asian racism over the course of this span of time.

Importantly, this study examined and analyzed responses from AANHPIs collectively. We recognize that AANHPIs represent a diverse diaspora of ethnic communities, and the aggregation of AANHPIs into a single group hides the unique historical, situational, and contextual experiences for each ethnic group. While the themes generated based on this diverse sample allude to potentially similar responses across different ethnic groups, it is possible that these themes may have different meanings by subgroups. Data aggregation among AANHPIs contributes to hidden experiences, and we recognize that this present study is aligned with this approach, despite our efforts to be as inclusive as many ethnic groups as possible. Moreover, AANHPIs possess other social identities, and the intersection of racial identity and these other identities may have contributed to unique response patterns that were not captured in this study.

### 3.2 Implications and Future Directions

Despite these limitations, this study embodies various principles of community psychology research (e.g., social justice and equity, diversity, strengths‐based approaches) by exploring how AANHPIs responded to anti‐Asian racism during the COVID‐19 pandemic. In addition, these findings have practical implications across multiple ecological systems and contexts. First, individuals working with AANHPIs should acknowledge and normalize the breadth of responses to anti‐Asian racism described in this study. For example, clinicians and community practitioners may reinforce AANHPIs' engagement in community philanthropy or civic participation as a method of healing. In addition, clinicians and community practitioners can use their skills in active listening, hold space for difficult emotions, and support communities in implementing programs to foster critical reflection and action (Chopra 2021). Second, collectives, organizations, and institutions can create and facilitate opportunities that promote AAHNPIs connectedness to one another through delving into cultural heritage. Displayed artwork can show linked historical connections, potlucks can encourage sharing of traditional recipes, events can center teaching of cultural practices, and book clubs or media nights can increase learning about AANHPI histories. Organizations should also consider opportunities that promote action and advocacy across marginalized groups, such as those that build solidarity between AANHPI and Black communities. This may include sharing knowledge about the history of AANHPI and Black solidarity, as well as providing suggestions on how to address anti‐Blackness with family members that emphasize cultural values of maintaining family harmony, respecting elders, and minimizing shame (Chopra 2021; David 2020). Third, policymakers have the power to financially invest in community‐based programs that support these responses and promote critical consciousness in AANHPI communities. For example, community scholars developed an intervention for Filipina American that uses art, poetry, and performances to promote personal reflection of oppression (Tintiangco‐Cubales and Sacramento 2009). Such programs could be elevated to increase their reach and scale.

This study also illuminates numerous areas for future investigations. First and foremost, research is needed to investigate responses to anti‐Asian racism by different groups (e.g., by ethnicity, gender, age, sexuality, nativity, etc.) and explore how the intersectionality of identities influences responses. For instance, some responses may be more contextually adaptive for first‐generation immigrants in rural areas, and some responses may differ for South Asians and East Asians who have different experiences of racialization in the United States and may have been impacted by COVID‐19‐related racism more in the form of xenophobia or Islamophobia (Cheng et al. 2021; Hussain 2020). Indeed, experiences across many diverse Asian ethnic groups vary due to colorism, religion, politicization, and more (Cheng et al. 2021; Lee and Thai 2015) and future investigations may be informed by a priori hypotheses based on historical and sociocultural differences. Future research is also needed to explore the relationship between responses and mental health and well‐being outcomes. For instance, research may continue to explore specific aspects of critical consciousness as it relates to anti‐Asian racism or White supremacy broadly and its association with well‐being in AANHPIs (Maker Castro, Du, et al. 2022; Lee 2022). Future research may explore how participants' degree of perceived impact by anti‐Asian racism is connected to their response (Yu et al. 2024). Based on associations between the model minority myth and experiences of discrimination, future research may also examine how endorsement of the model minority myth could be related to how AANHPIs responded to COVID‐related anti‐Asian racism. Finally, future research may leverage these responses to inform clinical assessment and intervention. For instance, these responses may inform the development of measures that assess AANHPIs collective responses to stressors. Research can also develop, test, and implement adaptations of therapy interventions to include a module with culturally derived responses that can promote individual and collective well‐being.

## 4 Conclusion

This qualitative study aimed to explore how AANHPIs responded to anti‐Asian racism during the COVID‐19 pandemic. The sample represented a diverse group of AANHPI adults, who had a shared racial identity as well as heterogenous experiences of and in response to anti‐Asian racism. We asked AANHPIs to describe their responses to anti‐Asian racism and three main themes and eight subthemes were identified. Responses fostered a sense of cultural authenticity, included extending care of others, and led to advocacy and activism for AANHPIs and other marginalized communities. This study characterizes AANHPIs' response to anti‐Asian racism through a lens of strength and resilience, and points to clinical and community implications for preventing and healing from racial oppression. For instance, clinicians can affirm the heterogenous responses that AANHPIs may have, organizations can facilitate events that promote critical consciousness, and policymakers can designate resources to support AANHPI community programs. There are also important directions for future research, such as by examining responses with a lens of disaggregation and by examining the association between responses and long‐term well‐being outcomes. We hope that these narratives inspire others to learn from culturally derived responses to healing and can inform efforts to mitigating the negative impacts of racism across the AANHPI diaspora.

## Author Contributions

**Julie K. Nguyen:** conceptualization, formal analysis, investigation, methodology, project administration, writing. **Wendy Chu:** conceptualization, data curation, formal analysis, funding acquisition, investigation, methodology, project administration, supervision, writing. **Stephanie H. Yu:** formal analysis, investigation, methodology, writing. **Andrea C. Ng:** conceptualization, data curation, formal analysis, investigation, methodology, writing.

## Ethics Statement

All study procedures were in accordance with the ethical standards of the Institutional Review Boards of the University of South Carolina.

## Consent

Informed consent was obtained from all participants.

## Conflicts of Interest

The authors declare no conflicts of interest.

## Data Availability

Data may be made available by contacting the corresponding author.
